# Evidence in Support of Potential Applications of Lipid Peroxidation Products in Cancer Treatment

**DOI:** 10.1155/2013/931251

**Published:** 2013-12-04

**Authors:** Omotayo O. Erejuwa, Siti A. Sulaiman, Mohd S. Ab Wahab

**Affiliations:** Department of Pharmacology, School of Medical Sciences, Universiti Sains Malaysia, 16150 Kubang Kerian, Kelantan, Malaysia

## Abstract

Cancer cells generate reactive oxygen species (ROS) resulting from mitochondrial dysfunction, stimulation of oncogenes, abnormal metabolism, and aggravated inflammatory activities. Available evidence also suggests that cancer cells depend on intrinsic ROS level for proliferation and survival. Both physiological and pathophysiological roles have been ascribed to ROS which cause lipid peroxidation. In spite of their injurious effects, the ROS and the resulting lipid peroxidation products could be beneficial in cancer treatment. This review presents research findings suggesting that ROS and the resulting lipid peroxidation products could be utilized to inhibit cancer growth or induce cancer cell death. It also underscores the potential of lipid peroxidation products to potentiate the antitumor effect of other anticancer agents. The review also highlights evidence demonstrating other potential applications of lipid peroxidation products in cancer treatment. These include the prospect of lipid peroxidation products as a diagnostic tool to predict the chances of cancer recurrence, to monitor treatment progress or how well cancer patients respond to therapy. Further and detailed research is required on how best to successfully, effectively, and selectively target cancer cells in humans using lipid peroxidation products. This may prove to be an important strategy to complement current treatment regimens for cancer patients.

## 1. Introduction

The physiological and pathophysiological roles of ROS have been a subject of research interests in the last few decades. Due to their short half-lives, quantification of ROS (either *in vivo* or *in vitro*) is a very challenging task. Consequently, the oxidation products of ROS in biological samples are of great interest because they are more stable and reflect the magnitude of oxidative stress [1]. One of such oxidation products is lipid peroxidation products which are formed when ROS attack polyunsaturated fatty acids (PUFAs) leading to membrane structural and/or functional damage [2]. Lipid peroxidation gives rise to the formation of highly reactive aldehydes which are extremely diffusible and attack or form covalent links with distant cellular components/targets [3]. Once ensued, lipid peroxidation is capable of self-propagating and initiating chain reactions [4]. In most cases, the reactions continue except they are terminated (e.g., by intervention with antioxidants such as vitamin E) or there is complete substrate utilization. Among the common lipid peroxidation products are malondialdehyde (MDA), 4-hydroxynonenal (HNE), and acrolein [5]. Others include isoprostanes (IsoPs) and neuroprostanes (neuroPs), which are oxidized products of arachidonic acid and docosahexaenoic acid, respectively [6]. These lipid peroxidation products covalently bind to histidine, cysteine, or lysine residues of protein through Michael addition resulting in structural alteration and impaired functions of these protein residues [7]. These aldehydes in turn propagate further attack on cellular membrane constituents, primarily lipids and proteins, to generate other end products of lipid peroxidation commonly known as lipofuscin-like pigments (LFPs) [8]. The role of lipid peroxidation is implicated in the pathophysiology of or associated with many chronic diseases such as Alzheimer's disease, diabetes mellitus, hypertension, and cancers in both animals and humans [9–12]. In spite of the detrimental biological effects of lipid peroxidation, this review presents evidence that suggests lipid peroxidation products could be utilized in the treatment of cancer.

## 2. Lipid Peroxidation Products

### 2.1. Malondialdehyde

Malondialdehyde is a highly reactive and toxic aldehyde formed as a consequence of peroxidation of PUFAs. MDA can also be produced from the breakdown of prostaglandin via the action of cyclooxygenase [13]. Other nonlipid precursors including carbohydrates and amino acids can also generate MDA [14]. Accumulation of MDA can alter the membrane permeability as well as impair fluidity of the membrane lipid bilayer [15]. MDA remains one of the most mutagenic lipid peroxidation products. It can react with deoxyadenosine and deoxyguanosine in DNA leading to the development of DNA adducts which are mutagenic [16, 17]. Malondialdehyde is a commonly used biomarker for the assessment of lipid peroxidation [18].

### 2.2. 4-Hydroxy-2-nonenal

4-Hydroxy-2-nonenal is a product generated following peroxidation of n-6 polyunsaturated fatty acids such as linoleic acid. Of all the aldehydes formed from the lipid peroxidation of PUFAs, HNE is considered the most important [19]. HNE, via Michael addition, is able to bind to cysteine, lysine, and histidine residues of proteins. These HNE-bound protein residues can impair normal protein structure and function [20]. HNE also reacts with many important cellular constituents such as nucleic acids, lipids, and vitamins as well as signaling molecules [21]. Such HNE interactions can interfere with normal cellular functions such as impaired glucose uptake at synapses and contributes to synaptic degeneration [22]. It can also cause loss of organelle functions such as microtubule dysfunction [23]. With these effects, accumulation of HNE can exert harmful effects on cellular functions and signaling and thereby elicit a number of pathological states including neurodegenerative disorders and cancers.

### 2.3. Acrolein

Acrolein is also one of the lipid peroxidation products of PUFAs. It is the most reactive of all the lipid peroxidation products [21]. In addition to PUFAs, it can be produced from partial burning of organic materials or fuel such as coal, petrol, and wood. It is also present in most types of smokes including cigarette smoke. Other sources of acrolein include cyclophosphamide bioactivation, threonine metabolism by myeloperoxidase of activated phagocytes, and overheated frying oils [5, 24]. Like HNE and MDA, acrolein is an electrophilic compound which rapidly interacts with or binds to key cellular nucleophiles and enzymes resulting in their depletion or inactivation [25]. It also reacts with nucleophilic sites in proteins and DNA, an important mechanism by which it exerts its cytotoxicity. Acrolein induces cell death, deterioration of cognitive function, and degeneration of hippocampal neurons, suggestive of its significant role in the pathophysiology of neurodegenerative diseases [26].

### 2.4. Isoprostanes

The isoprostanes, discovered in 1990, constitute a family of prostaglandin-like compounds formed as a consequence of free radical-induced oxidation of arachidonic acid (independently of cyclooxygenase) and later liberated from membrane phospholipids by phospholipases [27]. Compared to other lipid peroxidation products, IsoPs are more easily detected and chemically and metabolically stable in several biological samples such as urine, plasma, and tissues [28]. Accumulation of IsoPs can impair integrity, fluidity, and normal functions of membrane and is also associated with many pathophysiological disorders [29]. F2-IsoPs are among the well-researched IsoPs and considered better biomarkers of endogenous lipid peroxidation [30]. The measurement of F2-IsoPs is considered one of the most precise methods to evaluate oxidative stress and lipid peroxidative damage *in vivo* [28, 31]. Conversely, its use may be restricted in certain conditions with elevated concentrations of oxygen which suppress the formation of IsoPs [32].

### 2.5. Isofurans

Isofurans (IsoFs) belong to a class of compounds formed as a result of free radical-induced peroxidation of arachidonic acid. Similar to IsoPs, IsoFs are stable products, both chemically and metabolically [33]. However, unlike the IsoPs which are generated under low oxygen tension, the formation of IsoFs occurs or is favored at high oxygen concentrations [32, 34]. Therefore, isoFs may likely be important markers of oxidative stress in the brain as a result of its high oxygen consumption potential. As a result of the differences in oxygen tension required for the generation of IsoPs and IsoFs, concurrent quantification of both IsoPs and IsoFs is considered a better approach to evaluate oxidative damage in disorders or conditions with varying concentrations of oxygen such as anesthesia or surgery [35].

### 2.6. Neuroprostanes

The neuroprostanes (NeuroPs), also known as F4-isoprostanes, are products of free radical-catalyzed oxidation of docosahexaenoic acid (DHA). As a result of the elevated concentrations of DHA in neuronal membranes, neuroPs are regarded as important biomarkers for quantitative assessment of oxidative damage in neurons, cerebrospinal fluid, and brain tissues [36].

## 3. Cancer and Lipid Peroxidation Products

The incidence of and deaths due to cancers have risen vastly in the past years. These have been attributed to several factors. For instance, the global prevalence of lung cancer, which is the most predominant type of cancer, has been on the rise following increased worldwide cigarette smoking [37]. Other common factors linked to the increased cancer incidence and deaths include lifestyle patterns (such as physical inactivity, obesity, and alcohol consumption) and genetic disposition [38]. Research has linked ROS to the pathogenesis of many chronic diseases including cancers. While elevated levels are detrimental, moderate levels of ROS can be beneficial by serving as a second messenger in cell signaling [39]. It is, therefore, important to maintain redox homeostasis. The mechanisms of increased ROS formation in cancer remain poorly understood. Mitochondrial mutations or dysfunction, which are frequently observed in cancer patients, may play a role [40, 41]. Increased ROS formation due to mitochondrial mutations is caused by impaired electron transfer which results in leakage of electrons and subsequent generation of superoxide radical and other ROS [42]. Other factors implicated in the increased formation of ROS in cancer cells include oncogenic initiation, abnormal metabolism, and enhanced activity of inflammatory cytokines [43, 44].

Many forms of cancers are associated with enhanced ROS production resulting in lipid peroxidation. Plasma MDA levels have been found to be considerably higher in patients with lung cancer [45], breast cancer [46], colorectal cancer [47], and prostate cancer [48] than in healthy controls. Increased MDA levels have also been reported in patients with other forms of cancers such as laryngeal, oral cavity, and gastrointestinal tract cancers [49, 50]. Besides MDA, elevated levels of urinary IsoPs have been associated with breast or lung cancer risk [51]. Urinary IsoPs excretion was observed to be markedly higher in patients with prostate cancer than in the controls [52]. A similar increase in urinary IsoPs excretion was reported in gastric cancer patients [53]. These data clearly indicate that lipid peroxidation products are elevated in most forms of cancers and implicate the role of lipid peroxidation products in the etiology or progression of cancers.

## 4. Lipid Peroxidation Products and Cancer Growth Inhibition

Polyunsaturated fatty acids (PUFAs) of cell membrane are highly susceptible to lipid peroxidation. Hence, incorporating or increasing the PUFAs content of cancer cells may predispose these cells to enhanced lipid peroxidation. Besides, PUFAs in combination with anticancer agents such as doxorubicin and arsenic trioxide have been shown to elicit synergistic cytotoxic effect in various cancer cells such as breast, colon, cervical, pancreatic, and renal cancer cells [54–56]. A recent study showed that PUFAs augmented the sensitivity and cell death of leukemic cells to anticancer agents such as doxorubicin, vincristine, and fludarabine [57]. In addition to enhancing the antitumor effect of anticancer drugs, lipid peroxidation products may also be used as an adjunct in radiotherapy. A study found that omega-3 PUFAs enhanced the sensitivity of human colorectal adenocarcinoma cells to radiation in a dose-dependent manner [58]. In all these studies, increased cytotoxicity was mediated via enhanced formation of lipid peroxidation products such as MDA and ROS or via markedly reduced intracellular concentrations of glutathione and activity of glutathione S-transferase (an important enzyme that scavenges lipid peroxidation products) in cancer cells [57, 58]. This synergistic effect was markedly inhibited by antioxidants or lipid peroxidation inhibitors, such as N-acetylcysteine, lipoic acid, and *α*-tocopherol, and thereby corroborate the role of lipid peroxidation products [54–56].

Of late, reports indicate that lipid peroxidation products may serve as a therapeutic tool to induce death of proliferating tumor cells. In one of such studies, acrolein was found to exert cytotoxic effect in lung carcinoma and glioblastoma cells. This cytotoxicity was suppressed by lipid peroxidation inhibitors or antioxidants such as vitamin E, ebselen (a glutathione peroxidase mimic), and selenite [59]. In addition to its inhibiting effect on cancer growth, lipid peroxidation products in combination with antitumor agents or cancer therapy may exert additive or synergistic effect. Acrolein combined with TNF-related apoptosis-inducing ligand (TRAIL) potentiates TRAIL-induced apoptosis via downregulation of Bcl-2 expression and ROS-dependent upregulation of TRAIL death receptor 5 in human renal cancer cells [60]. Similarly, HNE in combination with panobinostat, a histone deacetylase inhibitor (HDACI), exerts greater inhibition of PC3 prostate cancer cell proliferation. The combination also induces greater G2/M arrest, DNA damage, and cell death in prostate cancer cells [61]. These findings reveal that lipid peroxidation products have antitumor activity and can also potentiate the cytotoxicity of anticancer agents and radiotherapy. Recent studies indicating that many investigational antitumor agents (such as elesclomol, costunolide, and deltonin) exert their anticancer effect via selective mitochondrial ROS induction in cancer cells provide additional evidence in support of the potential role of lipid peroxidation products in cancer growth inhibition [62–64].

The potential role of lipid peroxidation products in cancer growth inhibition has also been demonstrated in rodents. A study which investigated the effect of diets comprising varied amounts and types of fat in female athymic nude mice implanted with human breast carcinoma cells found that the mice fed fish oil diets had markedly lower mean volume of human breast carcinoma than those fed corn oil diets [65]. It was also observed that tumor lipid peroxidation product levels were significantly increased only in the fish oil diets-fed mice [65]. These data suggest that the reduced breast carcinoma volume observed in mice fed fish oil diets could be attributed to elevated levels of tumor lipid peroxidation products in the same mice. Addition of antioxidants to the fish oil diets significantly decreased the level of tumor peroxidation products and increased tumor volume. On the other hand, addition of ferric citrate (a potent inducer of lipid peroxidation) to the fish oil diets resulted in markedly increased levels of tumor lipid peroxidation products as well as diminished tumor volume [65]. Similarly, dietary fish oil supplementation in athymic mice implanted with MX-1 human mammary carcinoma was accompanied by tumor growth inhibition as well as increased tumor lipid peroxidation and protein oxidation products [66]. These data convincingly indicate that dietary fish oil inhibits the growth of breast carcinoma in nude mice via increased formation of tumor lipid peroxidation products.

In nude mice implanted with DLD-1 human colon cancer cells, *α*-eleostearic acid (*α*-ESA) supplementation induced apoptosis (enhanced DNA fragmentation) via increased formation of lipid peroxidation products [67]. The addition of an antioxidant led to suppression of lipid peroxidation products and apoptosis, indicating that the antitumor effect of *α*-ESA is mediated via accumulation of lipid peroxidation products [67]. A recent study also provides further evidence in support of the antitumor effect of lipid peroxidation product (HNE) in rodents. The authors found that blockade of mercapturic acid pathway-mediated elimination of HNE resulted in total remission of human cancer xenografts in nude mice [68].

## 5. Other Potential Applications of Lipid Peroxidation Products in Cancer Treatment

In addition to its inhibitory effect on cancer growth, available evidence suggests that the levels of lipid peroxidation products may reflect cancer severity. In patients with carcinoma of the oral cavity and oropharynx, it was found that MDA levels in patients with T3-4 tumors were markedly higher than in those with T1-2 cancers [69]. The authors also found that patients who exhibited recurrence had significantly higher MDA levels than those with complete remission [69]. Similarly, Chole and colleagues found that serum MDA levels in patients with oral precancer were much lower than in those with oral cancer, and it was observed to be the lowest in the controls [70]. Increasing levels of lipid peroxidation biomarkers including MDA and HNE have also been observed in patients suffering from other types of cancers (such as myeloid leukaemia, gastric, and breast cancers) as the disease progressed [71–73]. Lipid peroxidation products, therefore, could serve as a marker of initial stages of tumor development, progression, and metastasis as well as predicting the chances of cancer relapse.

The levels of oxidative stress markers such as lipid peroxidation products may also reveal the effectiveness of cancer therapy or surgical intervention. Reduced MDA levels have been demonstrated postoperatively in lung cancer patients following removal of cancer-associated parenchyma [74]. A similar finding (significantly reduced MDA levels) has also been reported in surgically treated colorectal cancer patients (both early and advanced stages) [75]. Hence, these findings suggest that, by comparing data obtained before and after cancer treatment, the levels of lipid peroxidation products could be used to monitor the progress and/or evaluate the effectiveness of therapy or surgery in patients with cancers.

## 6. Potential Mechanisms of Cancer Growth Inhibition by Lipid Peroxidation Products

In the last few years, the roles of several transcription factors have been implicated in the development of cancers. The p53 is a transcription factor which prevents free radical-induced gene mutations by detecting and getting rid of oxidatively damaged DNA [76]. Upon stimulation, p53 also induces a host of other genes which in turn cause cell cycle arrest and apoptosis [76, 77]. The loss of function of p53 family is known to contribute to cancer progression. In human neuroblastoma cell lines, HNE has been shown to inhibit proliferation, reduce S-phase cells, and induce apoptosis via upregulated expression of p53 tumor suppressor and target proteins [78].

The telomerase and telomerase reverse transcriptase (TERT) constitute an essential component of the telomerase complex which role is also implicated in the etiology and progression of cancer. Telomerase catalyzes the elongation of telomeres in DNA strands with the aid of telomerase complex [79]. This enzyme, therefore, plays an essential role in the transformation of senescent cells to potentially nonsenescent or immortal cells, which is of significant importance in tumorigenesis. HNE has been found to downregulate the expression and activity of TERT and telomerase, respectively, in human leukemic cell lines [80]. Similar to inhibitory effect of HNE on telomerase activity, downregulation of TERT expression and inhibition of cell proliferation have also been observed in colon carcinoma cells [81].

Besides inhibiting telomerase complex, lipid peroxidation products may modulate the expression of c-Myc/Mad/Max network. The c-Myc/Mad/Max network consists of transcription factors which interactions elicit activation or suppression of specific or target genes involved in cell-cycle progression, genomic instability, and tumor progression and expansion [82, 83]. For instance, the c-Myc which can activate telomerase complex (telomerase and TERT) is overexpressed in many forms of cancers [84]. The c-Myc also induces DNA damage and attenuates p53 function [85]. HNE has been shown to inhibit c-Myc expression while it upregulates Mad-1 expression. HNE also interferes with the DNA binding activity of c-Myc and Mad-1 to the hTERT promoter [80]. The ability of lipid peroxidation products such as HNE to modulate a number of transcription factors known to play vital roles in the pathophysiology or progression of cancer may be one of the mechanisms of its cytotoxicity in cancer cells. The summary of the effect of lipid peroxidation products on cancer cells is presented in Table 1.

## 7. Conclusions 

There is no doubt that ROS and lipid peroxidation play a role in cancer development. However, evidence also indicates that cancer cells require certain amounts of ROS for proliferation and survival. This, therefore, suggests strategies aimed at further increasing the levels of ROS and oxidative damage such as lipid peroxidation products may be deleterious to cancer cells and thus beneficial in cancer management. As highlighted in this review, compelling evidence shows that lipid peroxidation products exert antitumor effect and also potentiate the cytotoxicity of anticancer drugs and radiotherapy. Lipid peroxidation biomarkers could also serve as a diagnostic tool to predict the chances of cancer recurrence and be used to monitor the progress or effectiveness of therapy in cancer patients. The findings also reveal insights into the possible molecular mechanisms (via modulation of key cancer-related transcription factors) by which lipid peroxidation products may inhibit or suppress the growth of cancers. Selectively enhanced ROS formation and its suppressed elimination remain key viable options in exploring lipid peroxidation-mediated cancer cell death. At the moment, as a result of the double-edged sword property of ROS, one of the main challenges is how best to successfully, effectively, and selectively target cancer cells in humans using lipid peroxidation products. This is an area that requires further and detailed research as it may prove to be an important strategy to complement current treatment regimens for cancer patients.

## Figures and Tables

**Table 1:** A summary of the effect of lipid peroxidation products (alone or in combination with cancer therapy) on cancer cells.

Type of cancer or tumor cells	Lipid peroxidation products	Summary of key findings	Reference
Leukemic cells	MDA	Enhanced the cytotoxicity of doxorubicin, vincristine, and fludarabine on leukemic cells	[57]
Colorectal adenocarcinoma cells	MDA	Enhanced the sensitivity of colorectal adenocarcinoma cells to radiotherapy	[58]
Lung carcinoma and glioblastoma cells	Acrolein	Inhibition of tumor growth	[59]
Renal cancer cells	Acrolein	Potentiation of TRAIL-induced apoptosis; downregulated expression of Bcl-2; ROS dependent upregulation of TRAIL death receptor 5	[60]
Prostate cancer cells	HNE	Potentiation of inhibiting effect of panobinostat; augmented G2/M arrest; enhanced DNA damage and cell death	[61]
Breast and mammary carcinoma cells	MDA	Inhibition of tumor growth	[65, 66]
Colon cancer cells	MDA	Increased DNA fragmentation; induction of apoptosis	[67]
Neuroblastoma cells	HNE	Inhibition of cell proliferation; reduction of S-phase cells; induction of apoptosis; upregulated expression of p53 tumor suppressor and target proteins	[78]
Leukemic and colon carcinoma cells	HNE	Inhibition of cell proliferation; downregulation of TERT expression and telomerase activity; inhibition of c-Myc expression; activation of Mad-1 expression; interference with DNA binding activity of c-Myc and Mad-1 to TERT promoter	[80, 81]

MDA: malondialdehyde; HNE: 4-hydroxynonenal; ROS: reactive oxygen species; TERT: telomerase reverse transcriptase; TRAIL: TNF-related apoptosis-inducing ligand.
